# From nature to clinic: Quercetin’s role in breast cancer immunomodulation

**DOI:** 10.3389/fimmu.2024.1483459

**Published:** 2024-12-06

**Authors:** Liguang Fang, Dandan Gao, Tong Wang, Haijun Zhao, Yanan Zhang, Shijun Wang

**Affiliations:** ^1^ College of Traditional Chinese Medicine, Shandong University of Traditional Chinese Medicine, Jinan, Shandong, China; ^2^ The First Clinical Medical College, Shandong University of Traditional Chinese Medicine, Jinan, Shandong, China; ^3^ School of Nursing, Shandong University of Traditional Chinese Medicine, Jinan, Shandong, China; ^4^ Shandong Co-Innovation Center of Classic Traditional Chinese Medicine (TCM) Formula, Shandong University of Traditional Chinese Medicine, Jinan, Shandong, China

**Keywords:** Quercetin, breast cancer, immunotherapy, natural immunomodulator, tumor immune microenvironment

## Abstract

Immunotherapy has brought hope to many breast cancer patients, but not all patients benefit from it. Quercetin (Qu), a natural product found in various sources, has anti-inflammatory and anti-tumor properties. We conducted a review of the pharmacological research of Qu in regulating anti-tumor immunity *in vivo* and *in vitro*. Qu can directly regulate the local tumor microenvironment (TME) by enhancing the activity of immune cells which includes promoting the infiltration of T cells and natural killer (NK) cells, inhibiting the recruitment of myeloid-derived suppressor cells and tumor-associated macrophages. Additionally, Qu inhibits anaerobic glycolysis in tumor cells, thereby reducing the production and transport of lactic acid. It also suppresses tumor angiogenesis by targeting the vascular endothelial growth factor (VEGF) pathway and the vitamin D pathway. Furthermore, Qu can enhance the efficacy of immunotherapy for breast cancer by modulating the systemic microenvironment. This includes inhibiting obesity-related chronic inflammation to decrease the production of inflammatory factors, regulating the composition of intestinal microbiota, and intervening in the metabolism of intestinal flora. At the same time, we also address challenges in the clinical application of Qu, such as low absorption rates and unknown effective doses. In conclusion, we highlight Qu as a natural immunomodulator that enhances immune cell activity and has the potential to be developed as an adjunct for breast cancer.

## 1 Background

In 2020, breast cancer has surpassed lung cancer to become the most common malignant tumor in the world, and it ranks the fifth in the total number of cancer deaths (1).With the continuous development of treatment for breast cancer, especially the emergence of immunotherapy, the survival time of patients has been greatly prolonged. However, not all patients can benefit from immunotherapy (2), one of the main reasons is the immunosuppressive tumor microenvironments (TME).

There are many studies have shown that natural products have a regulatory effect on the tumor immune microenvironment (3), which can well improve the efficiency of immunotherapy. Quercetin (Qu) is a flavonoid from a wide range of sources, mainly derived from vegetables, coffee, tea and other plants, which has the effect of anti-tumor, anti-inflammatory, antioxidant (4). In addition to treating metabolic related diseases, Qu can directly act on breast cancer cells to promote the apoptosis by regulating the oxidative stress, ferroptosis, fat metabolism, aromatase promoter activation and miRNA (5–9). The study for Qu directly acting on tumor cells has been fully summarized and reviewed in previous reviews (10, 11) (**Table 1**). However, in the era of immunotherapy, it is still lacking to summarize the mechanism of Qu to treat breast cancer from the perspective of regulating the immune microenvironment. In order to improve the efficiency of immunotherapy for breast cancer and promote the clinical application of Qu as immunotherapy adjuvant, it is necessary to comprehensively summarize the mechanism of Qu regulating the immune microenvironment of breast cancer. Therefore, this paper reviews the mechanism of Qu regulating immune microenvironment. The articles we reviewed were characterized by clear dosing intervals, reasonable control group design, and accurate index observation, excluding unverified dummy studies and other studies that did not meet the criteria. In addition, we also discussed the future research directions and the challenges in the clinical application of Qu as an adjuvant for breast cancer immunotherapy.

**Table 1:** Qu directly inhibits breast cancer cell proliferation (nearly 5 years).

Type of treatment	Cancer cell line	Effects observed	References
Qu	MCF-7	The viability of stem cells (CD44+/CD24-) was reduced	(12)
Qu and silica nanoparticles	MCF7	Inhibition of MCF-7 cells proliferation and increasing of apoptosis by Quercetin nanoparticles	(13)
Vanadium and Qu	MCF7	The expression of p53, caspase-3 and caspase-9 in tumor cells was inhibited	(14)
Qu	MDA-MB-231	Inhibition of proliferation and invasion of breast cancer stem cells/Reduction of ALDH1A1, CXCR4, EpCAM and MUC1 protein levels/Induction of MDA-MB-231 cell cycle arrest in G2/M phase	(15)
Qu	MCF-7/MDA-MB-231	Down-regulating the expression of MMP-2, MMP-9 and VEGF proteins/Decreasing the protein levels of pyruvate kinase M2 (PKM2), glucose transporter 1 (GLUT1) and lactate dehydrogenase A (LDHA)/Inhibiting lactate production/Inhibiting glucose uptake/Inactivating the AKT-mTOR pathway	(16)
Qu	MCF-7和MDA-MB-231	Induced cell damage caused by ROS and inhibited the proliferation of tumor cells.	(17)
Sodium butyrate and Qu	MCF-7	Inhibit cell proliferation and decrease expression levels of ANXA5, ROS and mRNA	(18)
Qu	MCF-7	Decreased expression of CDK6/Reduce the viability and colony-forming potential of cancer cells/Decreased ROS production and CDK6 expression to induce apoptosis.	(19)
Quercetin 3-O-beta-D-galactoside pyranoside	MCF-7/4T1	B-cell lymphomato-2 (Bcl-2) and X-linked apoptosis inhibitor (XIAP) levels were decreased, while Bax and lytic caspase-3 levels were increased/The production of ROS (ROS) is reduced, thereby inhibiting the activation of the NF-κB signaling pathway.	(20)
5-Fu and Qu	MCF7	The expression of Bax and p53 genes and the activity of caspase-9 were increased and the expression of Bcl2 gene was decreased to improve apoptosis.	(21)
Qu	MCF7	Decreased Lef1 expression/Re-sensitized tumor cells to docetaxel/Downregulated ABCG2, Vim and Cav1 expression.	(22)
Qu	MDA-MB-231	Inhibition of IGF1R and its downstream kinases Akt and Erk1/2 activation/Inhibition of epithelial-mesenchymal transformation (EMT) transcription factors Snail and Slug expression in human MD-MB-231 breast cancer cells (TNBC cell line).	(23)
Qu	MCF7, MDA-MB-231, BT549, T47D and 4T1	Inhibition of cancer cell proliferation, migration and colony formation/Inhibition of matrix metalloproteinase signaling	(24)
Qu	BALB/c	Decreased intracellular mitochondrial respiration and glycolysis, limiting the widespread production of ATP	(25)
Qu	MDA-MB-231 和 MDA-MB-468	Inhibition of cytoplasmic HuR protein	(26)
5-Fu and Qu	MDA-MB-231	Decreased the mobility of MDA-MB-231 cancer cells and the expression of MMP-2 and MMP-9 genes.	(27)
Qu	MCF-7	Increased expression levels of DNA damage markers, H2AX and 8-OH-dG (*P* < 0.05)/Decreased the expression levels of DNA repair mediators RAD51, Ku70 and XRCC1 in cell lines.	(28)
Qu	MCF-7 和 MDA-MB-231	Inhibition of the expression of Hsp27 and Hsp70 and Hsp90.	(29)
Qu	MCF-7 和 MDA-231	Promotes TFEB expression and nuclear transcription, induces ferroptosis.	(30)
Qu and Nar	MCF-7	Decreased Bcl-2 gene expression and increased caspase 3/7 activity/Increased lipid peroxidation and decreased mitochondrial membrane potential (MMP), inducing cell apoptosis.	(31)
Qu and Tamoxifen	MCF-7 and MDA-MB-231	Regulation of the expression of genes involved in cell metastasis, cycle and apoptosis via the ER pathway.	(32)
Qu and Clonidamine	MCF-7	Block cell division/induce apoptosis of MCF-7 cells/Increase caspase level/Decrease MMP-2/-9 mRNA expression.	(33)
Qu	ZR-75-1, MCF-7, T47D and MDA-MB-231	Inhibit the growth, migration and invasion of BC cells/Inhibit the expression and activity of CYP3A4 in BC	(34)
Cisplatin+Qu	EMT6	Enhance the antitumor effects of cisplatin/decrease renal toxicity.	(35)

## 2 Qu regulates the local TME

**Figure 1:**
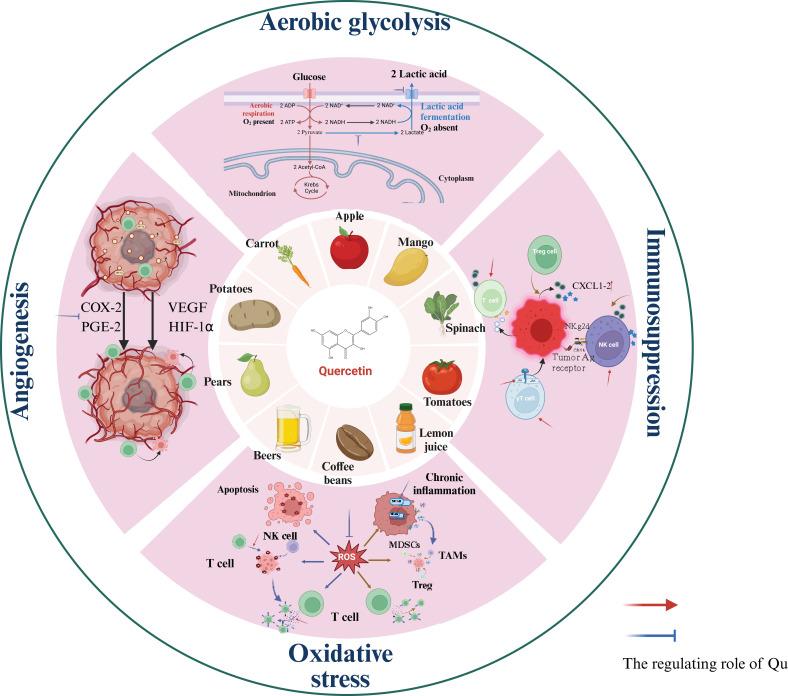
The source of Qu and its regulation in the local TME.

Due to the unique biological characteristics of tumor cells, the TME has the characteristics of acid-stage, hypoxia, ROS accumulation and so on, which ultimately leads to the immunosuppressive microenvironment. As a natural regulator, Qu can directly enhance the activity of effector T cells and NK cells (**Table 2**). Additionally, Qu inhibits the production of lactic acid by tumor cells through anaerobic glycolysis and reduces the expression of lactate transporters on the surface of tumor cells, thereby decreasing lactic acid accumulation in the TME. Qu also inhibits tumor angiogenesis not only via the VEGF pathway but also by downregulating vitamin D receptor expression and alleviating hypoxia within the TME. Finally, Qu can inhibit the accumulation of ROS, thereby mitigating their suppressive effects on anti-tumor immune cell activity (**Figure 1**).

**Table 2:** Qu regulates anti-tumor immunity.

Type of treatment	Cancer type	Effects observed	References
Folic acid and polyethylene glycol modified amphiphilic cyclodextrin nanoparticles for co-encapsulation of Rg3 and Qu (CD-PEG-PEG-FA.Rg3.QTN).	Colorectal cancer	Induce ROS lead to remission of immunosuppressive TME.	(36)
The fluorouracil and Qu were prepared by using the green material tea saponin (TS) as the binding molecule in a encapsulated nanoparticle (QU@FU-TS)	A549	QU@FU-TS promotes macrophage activation	(37)
Qu	MCF-10A, MCF-10AT, MCF-7 and MDA-MB-231	Regulate the expression of IFN-γ-R, p-JAK2, p-STAT1 and PD-L1 in the JAK/STAT1 signaling pathway, promoting the activation of γδ T cells,	(38)
Qu and Kaempferol	Colorectal cancer	Induce immune cell infiltration.	(39)
Qu and Propanolactone	Colorectal cancer	Stimulate host immune and induce memory tumor surveillance	(40)
Quercetin conjugates with iron ions (QFN).	4T1 cells	Promotes dendritic cell maturation and T cell activation	(41)
Qu	Hepatocellular carcinoma	Promote effect of NK cells and T cells.	(42)
Qu	Immunosuppressed rats	Reduce LPS-induced macrophage production of TNF-α, IL-1β and IL-6/IL-10.	(43)

### 2.1 Qu regulates the acidic TME

#### 2.1.1 Lactic acid production and immunosuppression

Lactic acid acts as the end product of glycolysis in tumor cells for energy production and promotes angiogenesis and immune evasion (44). Lactic acid produced by glycolytic activity is associated with poor prognosis with triple-negative breast cancer (TNBC) patients (45). Inhibiting the glycolytic process of tumor cells and reducing the content of lactic acid in TME can inhibit the activity of Myeloid-derived suppressor cells (MDSCs), promote the killing function of effector T cells (45) and enhance the anti-tumor immunity.

#### 2.1.2 Qu regulates the production and transport of lactic acid

Many studies have demonstrated that Qu can inhibit the glycolytic process in breast cancer cells. Compared with the control group, 30μM Qu significantly inhibited glucose uptake and lactate production in MCF-1/MDA-MB-231 cells, and decreased the levels of glycolytic-associated pyruvate kinase M2 (PKM2), glucose transporter 1 (GLUT1), and lactate dehydrogenase A (LDHA). Qu can inhibit tumor growth and metastasis by inhibiting the expression of VEGF and PKM2 *in vivo (* 16). In addition to inhibiting glycolysis, Qu also suppresses lactate transport in breast cancer cells. Monocarboxylate transporter (MCT) is associated with extracellular acidification and is highly expressed in MDA-MB-468 and Hs578T cells, but hardly expressed in MCF-7/AZ cells. It was observed that Qu only inhibited glucose consumption and lactic acid production in MDA-MB-468 and Hs578T cells, but had no effect on the metabolic behavior of MCF-7/AZ cells. This suggests that Qu inhibited lactic acid transport in breast cancer cells, and its effect was related to MCT1 (46).

### 2.2 Qu regulates angiogenesis

#### 2.2.1 Angiogenesis and immunosuppression

In TME, due to the over-expression of angiogenic factors in tumors, the tumor-associated vasculature is over-branched and disorganized, resulting in the reduction of oxygen content of TME and the promotion of immunosuppressive cell infiltration (47). Tumor-related vascular hyperplasia can further hinder the infiltration of effector T cells and promote T cell apoptosis (48).

#### 2.2.2 Qu suppresses tumor angiogenesis

Qu can directly inhibit tumor angiogenesis by acting on VEGF. MCF-7 cells were injected into the breast fat of female BALB/c nude mice and treated with Qu (34 mg/kg) for 21 days. The results showed that Qu decreased serum VEGF expression (*P* < 0.01) and microvascular density (*P* < 0.05). Qu treatment significantly inhibited tumor calcineurin activity (inhibition rate: 62.73%) and expression of nuclear factor of activated T cells (NFAT), a pathway critical in human breast cancer angiogenesis (49). Treatment of tamoxifen-resistant MCF-7 cells with six different flavonoids showed that treatment with 30μM Qu for 2 weeks was able to stably inhibit angiogenesis in breast cancer by inhibiting the expression of hypoxia-inducing factor-1α (HIF-1α) and activating protein-1 (AP-1), which were key transcription factors for VEGF gene transcription (50).

In addition to the VEGF pathways, Qu may also inhibit tumor angiogenesis through other mechanisms. Overexpression of the VDR can inhibit tumor angiogenesis (51). *In vivo* experiments using a mouse model of Ehrlich ascites cancer with Qu (50 mg/kg) intraperitoneal administration for 15 days showed significant inhibition of peritoneal neovascularization compared to the control group. Further *in vitro* experiments using human breast cancer cell lines (MDA-MB-231 and BT-474) demonstrated that Qu activates VDR to inhibit angiogenesis (52). Prostaglandin E2 (PGE2) binds to its receptor EP2 to directly promote tumor angiogenesis by enhancing endothelial cell survival and motility (53), while Qu significantly inhibits PGE2 production in breast cancer cells. In addition, Qu inhibited COX-2 promoter activation of MDA-MB-231 and MCF-231 breast cancer cell lines in a dose-dependent manner at 0-300μM and 0-200μM, and inhibited COX-2-mediated angiogenesis (54).

### 2.3 Qu regulates reactive oxygen species

Since cancer cells are under oxidative stress for a long time, they produce a large amount of ROS. ROS is a key driver of tumorigenesis, progression, metastasis and drug resistance. However, as the research progressed, the complex and diverse roles of ROS in TME have been gradually discovered (55).

#### 2.3.1 The dual role of ROS in anti-tumor immunity

In anti-tumor immunity, ROS is a double-edged sword. ROS can activate T cells and NK cells to kill cancer cells, and activated T cells and NK cells can increase the production of ROS, further promoting the recruitment and activation of both (56). On the other hand, elevated ROS can not only promote the infiltration and activation of immunosuppressor cells and inhibit the body’s anti-tumor immunity, including MDSCs, TAMs, and regulatory T cells (Tregs), but also inhibits T cell response by inhibiting the formation of TCR and MHC antigen complexes (57). Excessive ROS in T cells can reduce the levels of TCRζ chain and CD16ζ chain, block the activation of NF-kB, and lead to insufficient production of IFN-γ, TNF-α and IL-2 (58), thus inhibiting the activity of T cells. This contradictory effect of ROS may be related to the levels and different cell locations of ROS in TME. Continuous oxidative stress allows cancer cells to survive in high levels of ROS while maintaining cell viability, but can severely impair the activity of anti-tumor immune cells (59). The use of ROS as a therapeutic target for anti-tumor immunity largely depends on ROS levels and the tolerance of different cells to ROS. Targeting ROS in TME can be used as a therapeutic strategy to improve effector T cell function and enhance anti-tumor immunity (60).

#### 2.3.2 Qu regulates ROS production

Although there have been some drugs in clinical experiments confirmed that the antioxidant treatment could increase the effect of breast cancer immunotherapy (61). However, the selected drugs are still few and have some side effects.

Qu exhibits antioxidant properties, which can directly enhance the accumulation of ROS in tumor cells and induce their apoptosis (10). Treating MCF-7 cell lines with 25 μmol/mL Qu or Qu solid lipid nanoparticles (QU-SLN) for 48 hours, QU-SLNs significantly increased the level of ROS in MCF-7 cells and decreased the activity of antioxidant enzymes. Qu also increased the apoptosis and necrosis, but QT-SLNs showed more significant effects (62). It was found that after being treated with Qu and its water-soluble metabolites Qu 3’ -sulfate (Q3’s) and Qu 3-glucuronide (Q3G) at a dose of 25 µM for 48 hours, MCF-7 cell survival rates were 53.0% (*P* < 0.01), 55.6% (*P* < 0.01), and 65.1% (*P* < 0.05), significantly lower than the untreated group (100%). The cell survival rate decreased with the increase of drug concentration, and the inhibitory effect was dose-dependent, accompanied by the accumulation of ROS. The cytotoxic effects of Qu and Q3’S were similar to 5-fluorouracil at 100 µM (63).

Qu can directly reduce the ROS content in the TME (5) and alleviate the immunosuppression caused by excessive ROS levels. Compared with the ROS scavenger N-acetyl cysteine (NAC), Qu 3-o-β-D-galactoside pyranoside decreased ROS production in 4T1 cells with increasing concentration, functioning similarly to the NAC group. Both Qu 3-O-β-D-galactoside pyranoside and NAC treatment groups showed decreased IκBα phosphorylation and p65 expression levels. Qu 3-o-β-D-galactoside pyranoside inhibited the activation of the NF-κB signaling pathway by reducing intracellular ROS production (20), alleviating the immunosuppressive microenvironment. Sequential treatment of MDA-MB 231 cells, MDA-MB 468 cells, MCF-7 cells, and A549 cells with vitamin C and Qu reduced endogenous ROS production in a dose-dependent manner (*P*=0.027) and decreased ROS accumulation in the TME. The effective concentration of Qu ranges from 155.1 to 232.9μM (64).

## 3 Qu regulates the activity of immune cells

### 3.1 Qu regulates T cell activity

Qu can regulate the recruitment and functional activation of effector T cells. Qu increased CXCL1-2 expression in MDA-MB-231 and MCF-7 cells in a dose-dependent manner (*P* < 0.05 or *P* < 0.01) (20μM-80μM) (65). PD-L1 in TME can inhibit the function of T cells, and Qu activates T cells by inhibiting the binding of PD-L1/PD-1. The anti-PD-L1 antibody (200ng, Santa) was used as a control, while BSA (0.5μg/ml) served as a negative control. Qu (5μM) can inhibit PD-1/PD-L1 binding in HEK293 cells, and the inhibition rate reaching as high as 90%. In MDA-MB-231 and PBMCs xenograft mouse models, 60 mg/kg of Qu significantly inhibits the proliferation of tumor cells compared with the control group (0 mg/kg of Qu) and increases the protein levels of CD8, GZMB, and IFN-γ in tumor tissues (66). Gamma delta T cells (γδT cells) are a type of innate immune cell that plays an immunomodulatory role in the immune response to many infections and immune diseases (67). Qu concentration at concentrations of 2.5μM to 5μM can significantly promote the proliferation of γδ T cells *in vitro*. The killing ability of γδ T cells against breast cancer (MCF-10A > MCF-10AT > MCF-7 > MDA-MB-231) was increased after treatment with 5μM of Qu. When the ratio of γδ T cells to target cells (E/T) was 10:1, the killing effect was most pronounced, and the mechanism of Qu action was related to the JAK-STAT1 signaling pathway (38).

### 3.2 Qu regulates NK cell activity

In addition to effector T cells, Qu can influence the activity of NK cells. In a BALB/c mouse model constructed using 4T1 cells, the diet was supplemented with 1%, 2.5%, and 5% Qu, respectively, along with 50 mg/kg cyclophosphamide. The results showed that Qu combined with cyclophosphamide increased the activity of T cells and NK cells to a greater extent, and reduced the activity of Treg cells in the breast cancer microenvironment. Notably, 2.5% Qu had the most significant effect (68). This finding partially confirms the potential of Qu in regulating the chemotactic recruitment of immune cells. K562 and SNU1 cells were treated with 50 mM Qu for 30 hours, SNU-C4 cells with 20 mM Qu for 24 hours, and then the tumor cells were co-cultured with NK cells. The experimental results demonstrated that compared with tumor cells without Qu treatment, Qu increased the susceptibility of tumor cells to the toxic effects of NK cells. The cytotoxicity was reversed by the NKG2D receptor neutralizing antibody. Qu can effectively induce NKG2D ligands on the surface of tumor cells, thereby enhancing the tumor-killing effect of NK cells (69).

## 4 Qu regulates obesity-related chronic inflammation

Obesity is a complex chronic inflammatory disease that affects more than one third of the world’s population (70). Adipose tissue can induce chronic inflammation and negatively regulate the body’s immune surveillance function (71). Although drugs targeting obesity-related chronic inflammation have been widely used in the adjuvant treatment of obesity-related cancers, such as anti-inflammatory steroids and non-steroidal drugs, they often bring significant adverse effects in the course of long-term use, and sometimes have life-threatening consequences (72). Qu has strong anti-inflammatory activity and its side effects are relatively manageable, which has the potential to be developed as an adjuvant for immunotherapy against obesity-related cancers.

### 4.1 Obesity-related chronic inflammation inhibits anti-tumor immunity

In the UK, obesity elevates the risk of breast cancer among the postmenopausal population and results in a diminished response rate to anti-tumor therapies in breast cancer patients, ultimately contributing to a poorer prognosis (73). Additionally, obesity-related chronic inflammation and metabolic syndrome have been identified as independent prognostic factors that influence treatment response rates and the risk of mortality from breast cancer (74). Obesity-related chronic inflammation is systemic, but can reach the local TME through the circulatory system to exert immunosuppressive effects and suppress the effects of breast cancer immunotherapy (75), by inducing T cell exhaustion and immunosuppressive cell recruitment. In addition, chronic inflammation is frequently linked to tumor cachexia, which significantly impacts patients’ quality of life (76).

Obesity-related chronic inflammation can promote the infiltration of TAMs and CAFs and inhibit anti-tumor immune response. The crownlike structure (CLS), which is widely present in adipose tissue, is a structure in which macrophages clear dead adipose cells, but causes macrophages to be exposed to high levels of saturated fatty acids (77). Saturated fatty acids can activate TLR4 on the surface of macrophages, induce NF-κB signaling, and inhibit anti-tumor immunity (78). Obesity also increases the activity of NLRP3 in adipose tissue (79), which promotes the infiltration of MDSCs and TAMs, forms an immunosuppressive microenvironment (80). ASCs, as the main component of adipose tissue, can differentiate into cancer-associated fibroblasts (CAFs) (81, 82). In breast cancer, CAFs are able to suppress anti-tumor immunity through their metabolism and secretion of cytokines (83).

Obesity can lead to a decrease in both the number and diversity of T cells, as well as promote T cell exhaustion (84). In obese breast cancer patients, leptin in breast adipose tissue inhibits CD8+ T cell effector function by activating STAT3-fatty acid oxidation (FAO) and inhibiting glycolysis (85). Obesity also increases the concentration of CXCL1, promotes CXCR2 mediated Fas ligand (FasL) activation, and recruitment of MDSCs, leading to apoptosis of CD8+ T cells and decreased immunotherapy efficiency (86).

### 4.2 Qu treats obesity-related chronic inflammation

**Figure 2:**
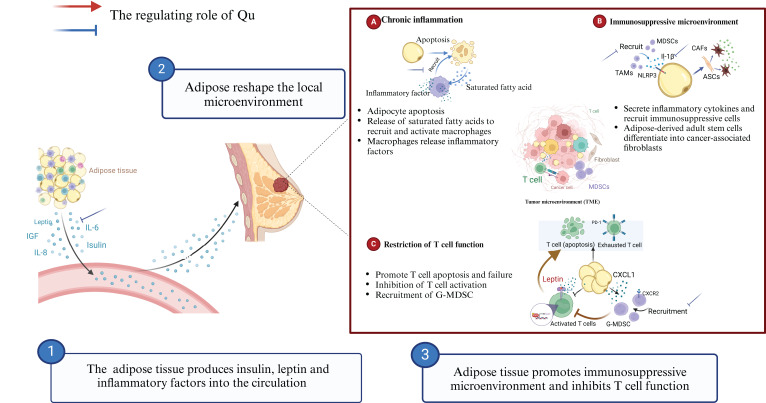
Qu modulates obesity-related chronic inflammation to enhance breast cancer immunotherapy.

Qu improves the prognosis of breast cancer patients by inhibiting obesity-related chronic inflammation, alleviating obesity-related metabolic syndrome, and increasing treatment sensitivity (**Figure 2**). Treatment of adipose-derived macrophages with Qu (0, 3, 10, or 30 mM) for 5 hours inhibited the expression of inflammatory genes, such as tumor necrosis factor (TNF)-α, interleukin (IL)-6, IL-8, and IL-1β compared with untreated macrophages (87). Qu (10 mg/kg) can reverse the obesity caused by a high-calorie diet *in vivo*. Compared with the high-calorie diet groups, phentermine groups, and orlistat groups, Qu can significantly inhibit the expression of pro-inflammatory genes such as IL-6, IL-1β, IL-18, and TNF-α, thereby alleviating the inflammatory state caused by obesity (88). When fed 50 mg/kg, 100 mg/kg, and 200 mg/kg respectively, Qu inhibited serum IL-6 production in a dose-dependent manner compared with high-fat diet (HFD) alone, but there was no difference in the reduction of serum TNF-α between groups. Compared with the use of metformin, Qu (300 mg/kg), can assist metformin in reducing the levels of serum IL-6 and TNF-α, relieving the inflammatory response of obesity, and has good safety, with no obvious organ toxicity (89).

Obesity-related chronic inflammation can lead to the infiltration of inhibitory immune cells, promote T cell exhaustion, and diminish the effectiveness of breast cancer immunotherapy. Qu can directly influence the metabolism of adipose tissue and inhibit the development of obesity-related chronic inflammation by reducing the production of inflammatory factors and the recruitment of TAMs. This has positive implications for the clinical application of immunotherapy. Dietary Qu (17 mg/kg) for 9 weeks induced white fat remodeling and decreased IL-6 and adiponectin levels in adipose tissue in obese mice (90). Qu inhibits the infiltration of macrophages in adipose tissue to relieve chronic inflammation. After 12 weeks of supplementation with 0.1% Qu (Purity ≥98%) on the basis of HFD, Qu significantly reduced the infiltration of macrophages in adipose tissue compared with HFD alone. Qu increased the number of M2-type macrophages while decreasing the levels of pro-inflammatory cytokines TNF-α, IL-6, and MCP-1. Qu activates AMPKα1/SIRT1 signaling to inhibit the polarization and inflammation of bone marrow derived macrophages *in vitro (* 91). In addition to feeding with a Western diet (high fat, high cholesterol, and high sucrose), 0.05% Qu was supplemented for 18 weeks. The results showed that Qu significantly inhibited the increase of serum TNF-α, leptin, and decreased the protein expression of NF-κB p65 in western diet mice. Immunohistochemistry showed that Qu inhibited Western diet-induced macrophage accumulation in adipose tissue (92).

In summary, the metabolism of adipose tissue frequently induces chronic inflammation, characterized by the accumulation of inflammatory factors and populations of white blood cells, which often inhibits the anti-tumor immune response. Tumor cells themselves also produce various chemokines to attract neutrophils, macrophages, myeloid-derived suppressor cells, and other immune cells. These cells, in turn, secrete a range of cytokines and cytotoxic mediators. Therefore, in cancer patients, regardless of obesity, chronic inflammation is prevalent and often adversely affects the response to tumor immunotherapy. Given the widespread nature of chronic inflammation in cancer patients, utilizing Qu as a routine clinical intervention to suppress chronic inflammation presents a promising treatment strategy. This approach may enhance the immunotherapy response rate and improve patient prognosis.

## 5 Qu regulates gut microbiota

The interaction between gut microbiota and the immune system can significantly affect the anti-tumor immune response. Therefore, the immuno-oncology-microbiome axis hypothesis was gradually formed to summarize the mechanism of gut microbiota in tumor pathogenesis and anti-tumor immunity (93). Qu can affect the anti-tumor immunity of the body by affecting the gut microbiota.

### 5.1 Gut microbiota regulate anti-tumor immunity

Many studies have demonstrated that the composition of intestinal flora significantly influences the effectiveness of breast cancer immunotherapy. Certain specific types of flora can even serve as biomarkers to predict the efficacy of this treatment. Qu has the ability to modulate the composition of intestinal flora, enhance its abundance, and increase the overall microbial diversity, thereby improving the effectiveness of immunotherapy. Additionally, the metabolites produced by these flora can impact the efficiency of immunotherapy by reshaping the immune microenvironment associated with breast cancer. Qu can also regulate the metabolism of intestinal flora, leading to an increased accumulation of beneficial metabolites, which in turn affects the efficacy of immunotherapy. Metabolites produced by gut microbiota get to local microenvironment of breast cancer through the circulatory system, and regulate key signaling pathways in cancer cells and various immune cells affecting the efficiency of anti-tumor immunotherapy (94). Butyrate, a metabolite of gut microbiota, improves the efficacy of anti-PD-1 immunotherapy by regulating TCR signaling in cytotoxic T cells (95). *Intestinal B. pseudolongum* enhances anti-tumor immunotherapy response by producing the inosine (96). Immunotherapy leads to a decrease in intestinal barrier function, and local inosine in the intestine is more likely to participate in the systemic circulation. Short chain fatty acids (SCFA) produced by *Escherichia* coli have cytotoxic effects on breast cancer cells and directly inhibit breast cancer cell proliferation (97). Gut microbiota and its metabolites can not only promote the immune response, but also inhibit the immune response of the body and promote the progression of tumors. In a C3-1-TAg mouse model of breast cancer, it has been observed that liver infection with *Helicobacter hepaticus*, a gut-resident bacterium, can induce breast cancer progression by promoting neutrophil recruitment and infiltration in TME, shaping an immunosuppressive microenvironment (98).

In addition to metabolites, extracellular polysaccharides or surface proteins in the structure of bacteria themselves can act as pathogen-associated molecular patterns (PAMPs), directly stimulating intestinal immune cells and inducing innate or adaptive immune responses to regulate anti-breast cancer immune responses. Exopolysaccharides produced by *Lactobacillus delbrueckii subsp*. bulgaricus OLL1073R-1 (EPS-R1) induce CCR6+CD8+T cells in mice and humans. In mice, ingestion of EPS-R1 enhanced the anti-tumor effects of anti-CTLA-4 or anti-PD-1 monoclonal antibodies against tumors expressing CCL20 (99). Immunostimulating exopolypolysaccharide (EPS) produced by *Lactobacillus delbrueckii ssp*. bulgaricus OLL1073R-1 enhanced activity of natural killer (NK) cell in mouse spleen cells and induced production of IFN-γ (100).

### 5.2 Qu regulates intestinal flora to promote anti-tumor immunity

**Figure 3:**
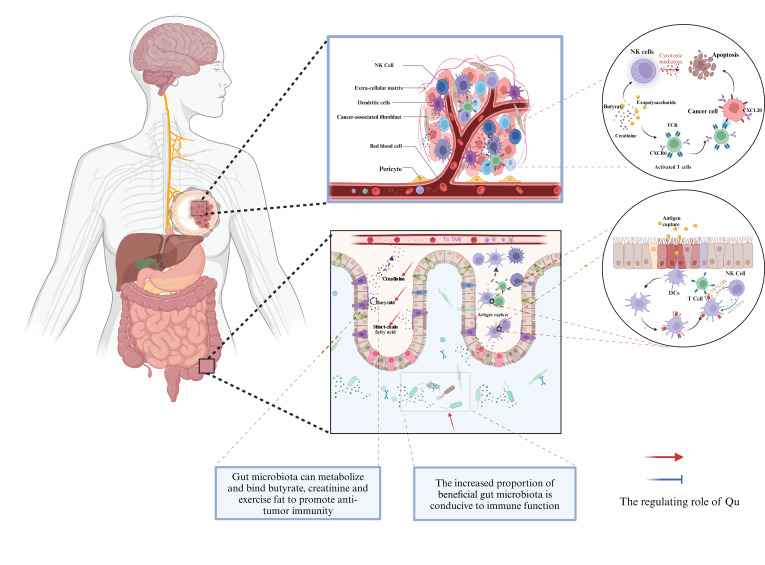
Qu regulates gut microbiota to enhance breast cancer immunotherapy.

Qu can not only directly inhibit the proliferation of tumor cells by regulating intestinal flora (**Table 3**) but also improve the efficiency of anti-tumor immunotherapy. Qu can regulate the proportion of intestinal flora and increase the number of beneficial flora (**Figure 3**). In the TNBC mouse model, the addition of 2.5% Qu to the diet significantly induced *Akkermansia* enrichment and increased the activation of effector T cells and NK cells compared with mice without Qu supplementation (68). In a mouse model of hepatocellular carcinoma, the combination of Qu and immune checkpoint inhibitors increased the abundance of *Firmicutes*, *Actinobacteria*, and *Vermilata* microbiota, as well as *Dubosiella* and *Acementia* at the genus level, and increased the expression of CD8a, CD4, CD11b, IL-10, and IFN-γ in the TME compared with Qu or immune checkpoint inhibitors alone (106). In a mouse model of colitis, additional supplementation with Qu (30 mg/kg) increased the numbers of *Bacteroides*, *Bifidobacterium*, *Lactobacillus*, and *Clostridium*, and significantly decreased the numbers of *Clostridium* and *Enterococcus* (*P* < 0.05) (107). In the model of using antibiotics to disrupt the balance of intestinal flora, the experimental group added 0.2% Qu based on AIN-93G feeding, and the results showed that Qu increased the level of intestinal beneficial bacteria species. These included *Faecalibaculum rodentium* (103.13%), *Enterorhabdus caecimuris* (4.13%), *Eggerthella lenta* (4%), *Roseburia hominis* (1.33%), and *Enterorhabdus mucosicola* (1.79%). These bacteria can produce butyrate and reduce serum lipopolysaccharide and TNF-α levels (108). The mice fed with 0.05% Qu based on HFD lasted for 6 weeks. Compared with the mice in the HFD group, Qu significantly reduced liver fat and blood glucose levels. In feces, the relative abundance of *Akkermansia* was significantly increased (109).

**Table 3:** Qu treats tumors via gut microbiota.

Type of treatment	Cancer type	Effects observed	References
2,4,6-THBA and 3,4-DHBA (Bacillus glycini can be produced from Qu)	HCT-116	Inhibit the proliferation of tumor cells.	(101)
3,4-dihydroxyphenylacetic acid (3,4HPAA) (Qu microbiota derived metabolite)	Colorectal cancer	Increased intracellular and mitochondrial ROS production.	(102)
Alternating intake of beta-glucan and Qu	Colorectal cancer	Decreased TNF-α levels/Increased the relative abundance of Paracoides/Down-regulated three genes (Hmgcs2, Fabp2, and Gpt) associated with inflammation and cancer.	(103)
Qu and Probiotics (such as Bifidobacterium bifidum and Lactobacillus gardneri)	Colorectal cancer	Inhibit Wnt/β-catenin signaling pathway.	(104)
Quercetin and its metabolites from human gut bacteria	HCT-116, A549 and HeLa	Inhibit proliferation of HCT-116 cells, A549 cells and HeLa cells.	(105)

Qu can directly regulate the metabolites of intestinal flora to exert anti-inflammatory effects, thereby shaping an effective anti-tumor immune microenvironment (**Figure 3**). Through the antibiotic induced intestinal flora dysregulation mouse model, the experimental group was fed AIN-93G diet containing 0.2% Qu for 10 days. Through the analysis of mouse feces and serum metabolites, it was clear that isorhamnetin, a Qu methylated metabolite, was the main active component in serum. Compared with antibiotic intervention alone, Qu treatment was able to increase the diversity of intestinal flora (still lower than that of the group without antibiotics) and butyrate levels (110). Compared with HFD-fed mice, mice fed with 1% Qu for 16 weeks had lower body weight and total plasma cholesterol, reduced the ratio of *Firmicutes*, *Bacteroidetes* in the stool of HFD-fed mice and increased the production of short-chain fatty acids (SCFA) (111). Studies have shown that SCFA can improve the efficiency of breast cancer immunotherapy (112).

Based on the regulatory effects of Qu on intestinal flora, clinical treatment strategies can be influenced by the following three aspects. First, the sensitivity of intestinal flora richness can predict the efficacy of immunotherapy. A higher richness of intestinal flora often correlates with a better response to immunotherapy. Therefore, in the clinical application of immunotherapy, the bacterial flora of patients can be sequenced in advance to assess their microbial composition. Based on the results of microbiota sequencing, Qu can be administered beforehand to enhance the abundance of intestinal flora, thereby improving the response rate to immunotherapy. Second, the expression of beneficial bacteria often enhances the response to immunotherapy, including species such as *Ackermannia* and *Ruminococcus*.etc. Qu has been shown to improve the expression of these beneficial bacteria, thereby increasing the efficacy of immunotherapy. In addition to utilizing natural products, the incorporation of probiotics can also enhance the recruitment of beneficial bacteria in the gut. Third, some metabolites produced by intestinal flora can boost the effectiveness of immunotherapy. Qu can promote bacterial metabolism, making its application advantageous for increasing the accumulation of bacterial metabolites, which is beneficial for the clinical application of immunotherapy. Therefore, given Qu’s impact on intestinal flora, it has the potential to be developed as an adjuvant for tumor immunotherapy.

## 6 Comparison of the immunomodulatory effects of Qu and other natural products

Numerous *in vivo* and *in vitro* experimental studies have demonstrated that natural products possess a positive regulatory effect on the immune system and can enhance tumor control. Due to their diverse sources and relatively low toxicity, natural products and their derivatives have become essential in the design and development of anti-tumor drugs (113).

Similar to the role of Qu, the immunomodulatory effects of various natural products and their derivatives extend beyond direct cytotoxic effects, they also play a crucial role in reshaping the immune microenvironment and inhibiting chronic inflammation. These natural products can stimulate immune cells, enhancing their activity, including CD8+ T cells, NKs, DCs, or inhibit the recruitment of immunosuppressive cells within the TME, such as MDSCs, Tregs, and TAMs, thereby blocking immunosuppression in the TME. Furthermore, in addition to their direct actions on the TME, the inhibition of chronic inflammation represents a significant mechanism through which natural products regulate anti-tumor immunity, including the suppression of signaling pathways such as NF-κB and JAK-STAT. Resveratrol is classified as a stilbene. It can inhibit the interaction between PD-1 and PD-L1, thereby enhancing the activity of cytotoxic T lymphocytes and improving the response rate to immunotherapy (114). Vanillic acid, an aromatic phenolic compound, induces macrophages to polarize into the M1 phenotype by activating the IL-6R/Janus kinase (JAK) signaling pathway, which enhances anti-tumor immunity (115). Ursolic acid, a triterpenoid compound, reduces the recruitment of MDSCs and Tregs in tumor tissue, thereby reshaping the immunosuppressive microenvironment associated with breast cancer (116). Fucose gum, a sulfated polysaccharide, enhances both adaptive and innate immune responses by boosting the activity of T cells, macrophages, DC, and NK cells (117). Anthraquinone (AQ) mitigates oxidative stress and mitochondrial damage in MCF-7 cells and inhibits the proliferation of MCF-7 breast cancer cells in a concentration-dependent manner (118).

Many natural products possess the ability to regulate anti-tumor immunity. However, the varying mechanisms of action among these products, along with the unclear effective dosages and low bioavailability *in vivo*, complicate the execution of clinical trials involving natural active compounds. Consequently, the efficacy of different natural products in regulating anti-tumor immunity has not been thoroughly investigated. Nevertheless, combining various natural products appears to enhance the effects of individual compounds. Specifically, the combination of curcumin, resveratrol, and Qu (RCQ) has been shown to reshape anti-tumor immunity in TNBC by modulating macrophage polarization, increasing the recruitment of effector T cells, and shifting the immune balance toward an immune-activated state (119).

## 7 Adjuvant application of Qu in tumor therapy

Qu has certain cytotoxic effects and demonstrates a synergistic effect when combined with chemotherapy in clinical practice. The concurrent use of Qu and docetaxel significantly inhibited cell growth and induced apoptosis in the MDA-MB-231 cell (120). The combination of docetaxel (7nM) and Qu (95μM) produced the most substantial synergistic effect, with a value of 34,268,250. In estrogen receptor-positive breast cancer, low concentrations of Qu (1-20μM) significantly inhibited tamoxifen-induced proliferation of MCF-7 cells, while higher concentrations of Qu (≥50μM) synergistically enhanced tamoxifen-induced apoptosis (32). Qu has also been shown to improve the efficacy of the gemcitabine and doxorubicin combination by downregulating the expression of HIF-1α and increasing the expression level of the apoptosis regulator p53 (121). In breast cancer cells, the combination of Qu and doxorubicin further enhanced the antitumor effect of doxorubicin (122). Qu inhibits YAP expression and its translocation to the nucleus, leading to the restoration of the Hippo pathway, which inhibits cancer progression and increases cisplatin sensitivity, thereby facilitating effective synergistic chemotherapy applications (123).

Qu and its derivatives can effectively target the regulation of the TME and support the advancement of immunotherapy. By modulating the expression of p-JAK2, p-STAT1 and PD-L1 in the JAK/STAT1 signaling pathway, Qu enhances the regulation of γδ T cells and improves the cytotoxic activity of immune cells against breast cancer (124). Qu and iron ions (QFN) were combined to create a multifunctional nano-photosensitizer. Qu released by QFN can decrease the expression of PD-L1 in tumor cells by inhibiting the phosphorylation of JAK2 and STAT3, thereby enhancing anti-tumor immunity (41). The combination of folic acid (FA) and polyethylene glycol (PEG) modified amphiphilic cyclodextrin nanoparticles (NPs) encapsulated ginsenosides and Qu into a nano-preparation (CD-PEG-FA.Rg3.QTN) along with anti-PD-L1 antibodies, significantly improves the efficacy of immunotherapy for colorectal cancer and prolongs the survival rate of animal models (36).

## 8 Discussion

Qu, as a natural product, is widely present in many fruits and vegetables. However, there are several pressing issues that need to be addressed to enhance its potential use as a drug in clinical practice.

The low oral bioavailability of Qu hinders its ability to exert effective biological activity *in vivo*. Many researchers have made significant progress in enhancing the bioavailability of Qu by coupling it with nanoparticles (125). Compared with free Qu treatment, AuNPs-Qu-5 treatment reduced HUVECs cell viability in a dose-dependent manner, inhibiting *in vitro* angiogenesis by suppressing the formation of capillary tubes in human umbilical vein endothelial cells (HUVECs) at 50μM. Gold nanoparticle-conjugated Qu (AuNPs‐Qu‐5) downregulated VEGFR-2 protein expression in HUVECs. In a DMBA-induced breast cancer rat model treated with free Qu and AuNPs‐Qu‐5, AuNPs‐Qu‐5 significantly suppressed tumor growth and prolonged the survival time of tumor-bearing rats compared with free Qu-treated rats (126). After the engineering modification of Qu, its function of promoting T cell activation becomes more significant. The Qu polyethylene glycol co-nano module (P-Qu-MTX-Fe) can effectively inhibit the recurrence of primary and metastatic breast tumors by activating anti-tumor immune responses mediated by CD8+T cells (127). When encapsulated in phospholipid complexes, Qu can be delivered directly into cells, significantly enhancing its bioavailability (128, 129). In addition to the structural modifications of Qu, extensive *in vivo* studies on Qu’s secondary metabolites and the development of their applications represent effective strategies to enhance Qu’s utilization rate. Many researchers have modified the structure of Qu by incorporating nanomaterials to enhance its absorption rate and biological activity (130), leading to some success in modulating anti-tumor immunity. Future research could focus on developing novel drug combinations that support anti-tumor immunity by combining immune checkpoint blockade (ICB) with Qu.

Due to the numerous targets of Qu immunomodulatory action, its low oral bioavailability, and the uncertainties surrounding effective dosages and potential toxic side effects, it is difficult to carry out clinical trials to investigate its anti-tumor effects poses significant challenges. Consequently, there is currently no clinical evidence to support the use of Qu for the prevention or treatment of cancer in humans. However, as a naturally occurring dietary compound, extensive animal studies have confirmed its efficacy and safety, indicating its potential for development as an adjuvant in tumor immunotherapy. Qu can be used as a dietary supplement to assist in the immunotherapy of tumors, but the optimal and safe dose for the tumor patient population remains to be studied (131). *In vivo* experiments in mice have shown that high-purity Qu, as an additive in the range of 0.05%-0.5% added to HFD, can have a better anti-inflammatory effect, with no observed toxicity. However, most of these studies focused on obese mice or colitis mouse models. Tumor mice often experience an increased metabolic load due to tumor metabolism and metastasis, so the safety of Qu in tumor patients needs further study. Building on the research results of Qu in metabolic diseases, inflammatory diseases, and intestinal flora metabolism, future research should conduct extensive *in vivo* experiments and large-scale clinical trials to thoroughly understand its adjuvant effect on breast cancer immunotherapy and evaluate its safety. In addition, by employing current multi-omics research methods and leveraging artificial intelligence, we can comprehensively integrate and analyze the research data related to Qu. This analysis will focus on its targets, effective dosages, and potential adverse reactions, thereby establishing a solid foundation for subsequent rigorous clinical trials. Ultimately, this will facilitate the clinical application of Qu.

In summary, this paper reviews numerous *in vivo* and *in vitro* studies to elucidate the mechanisms by which Qu modifies the immune microenvironment in breast cancer by modulating both local and systemic factors. The findings indicate that Qu, as a natural immunomodulator, has the potential to be developed as an adjuvant for breast immunotherapy. However, there remain several challenges that researchers and clinicians must address in order to expedite the development and application of Qu as an immunotherapy adjuvant.
